# Dataset of prefrontal transcranial direct-current stimulation to improve early surgical knot-tying skills

**DOI:** 10.1016/j.dib.2021.106905

**Published:** 2021-02-23

**Authors:** Ronak Patel, Harsimrat Singh, James Ashcroft, Adam J Woods, Ara Darzi, Daniel R Leff

**Affiliations:** a Department of Surgery & Cancer, Imperial College London, St Mary's Hospital Campus, 10th Floor, QEQM Building, Praed Street, London W2 1NY, United Kingdom; b Department of Clinical and Health Psychology, Center for Cognitive Aging and Memory, McKnight Brain Institute, University of Florida, Gainesville, FL, United States

**Keywords:** Transcranial direct-current stimulation, Psychomotor performance, Prefrontal cortex, Motor skills, operative surgical procedures

## Abstract

Transcranial direct-current stimulation (tDCS) has previously demonstrated promising effects in improving surgical performance with motor region stimulation [1], [2], [3], [4]. However, extensive prior research has revealed an important role of the prefrontal cortex in surgical skill development [5,6]. This article presents the data of a double-blind randomized sham-controlled trial investigating the effect of prefrontal tDCS on knot-tying performance [7]. Data was collected from an active (*n* = 20) and sham (*n* = 20) group across three blocks: pre-, online- (during) and post-tDCS. Group and block differences of knot-tying performance were analyzed using a Generalized linear mixed model and supported with a Friedman's test. Further sub-analyses were conducted to compare high vs. low skilled individuals and initial vs. last knots. Subjective workload was assessed after each block using a SURG-TLX questionnaire and side-effects of the tDCS block were recorded using an additional survey.

## Specifications Table

**Table:** 

Subject	Neuroscience: Behavioral
Specific subject area	Motor skill enhancement
Type of data	Tables
How data were acquired	Knot-tying assessment, Side-effect questionnaire, SURG-TLX questionnaire
Data format	Raw Analyzed
Parameters for data collection	A double-blind randomized sham-controlled trial investigated the effect of prefrontal tDCS on knot-tying performance and the associated subjective workload. Two groups received either active (*n* = 20) or sham (*n* = 20) prefrontal tDCS. Measures were recorded in three sessions: pre-, online- (during) and post-tDCS.
Description of data collection	For each session, knot speed, accuracy and security were measured by a single blinded assessor. Subjective workload was assessed using a SURG-TLX questionnaire. Side-effects of the online-tDCS session were also surveyed.
Data source location	Imperial College London, London, UK
Data accessibility	Raw data: DOI: https://doi.org/10.14469/hpc/7891 Supplementary material: DOI: https://doi.org/10.14469/hpc/7890
Related research article	J. Ashcroft, R. Patel, A.J Woods, A. Darzi, H. Singh, D.R. Leff. Prefrontal Transcranial Direct-Current Stimulation Improves Early Technical Skills in Surgery. Brain Stimulation. https://doi.org/10.1016/j.brs.2020.10.013

## Value of the Data

• This dataset presents the impact of prefrontal tDCS on surgical knot-tying performance, as well as the impact on subjective workload demands and the side-effect profile data.
• The data can be used by other researchers to explore the behavioral effects of tDCS delivered to the prefrontal cortex in a manual dexterity task.
• The dataset can be compared to similar tDCS studies in surgical performance to evaluate for the most efficacious tDCS assembly and stimulation parameters, whilst also understanding the relative side-effect profiles in this setting.

## 1 Data Description

The dataset reported here was collected from a double-blind randomized sham-controlled trial investigating the performance enhancing effects of prefrontal transcranial direct-current stimulation (tDCS) on surgical knot-tying performance [7]. The raw data can be found at DOI: 10.14469/hpc/7891. The dataset reported within this article consists of Table 1 which reports the median behavioral scores including knot-tying times, error subcomponent scores and overall performance scores, and with Table 2 which reports the SURG-TLX scores. Table 3 presents the statistical output of the generalized linear mixed model used to compare group and block differences and Table 4 provides supporting analysis of group (Mann Whitney U test) and block (Friedman test) differences. Table 5 represents further subgroup analysis comparing the median performance scores of high and low-skilled individuals and the initial and last knots in each group. Table 6 reports on the sensation responses to assess side effects of the tDCS blocks. Finally, the SURG-TLX and sensation surveys are both provided in the Supplementary material (DOI: 10.14469/hpc/7890).

**Table 1:** Median time and error scores with overall median performance score for each participant. Knot breakage not shown as no occurrences.

		Time			Accuracy			Gap			Slippage			Performance score		
Subject	tDCS	Pre	Online	Post	Pre	Online	Post	Pre	Online	Post	Pre	Online	Post	Pre	Online	Post
1	Active	39	45	37	0	0	0	1	0	0	0	0	0	11	10	23
2	Active	49	54	49	2	1	1	1	0	0	0	0	0	−16	−84	−7
3	Active	58	49	38	1	0	0	1	0	0	0	0	0	−6	1	18
4	Active	41	33	32	1	0	0	1	1	0	0	0	0	0	15	20
5	Active	45	44	41	1	1	1	0	0	1	0	0	0	3	3	1
6	Active	48	34	36	0	0	0	0	0	0	0	0	0	12	26	24
7	Active	46	45	40	1	1	1	0	0	0	0	0	0	3	−3	2
8	Active	44	35	32	0	0	0	0	0	0	0	0	0	16	24	28
9	Active	48	39	44	0	0	0	0	0	0	0	0	0	10	21	16
10	Active	41	43	35	1	0	0	0	0	0	10	0	0	−75	8	20
11	Active	57	49	39	1	0	0	1	1	1	20	20	20	−202	−185	−188
12	Active	51	49	49	1	2	1	0	0	0	0	0	0	−10	−9	−9
13	Active	54	46	44	0	0	1	0	0	1	0	0	0	−7	14	7
14	Active	39	34	36	0	0	0	0	0	0	0	0	0	21	26	24
15	Active	31	27	30	2	5	2	0	0	0	0	0	0	−2	−16	8
16	Active	43	45	35	0	0	0	1	1	0	0	0	0	7	1	16
17	Active	58	40	40	1	0	0	0	0	0	0	0	0	−9	20	20
18	Active	51	43	37	1	0	1	0	0	0	0	0	0	−20	−2	2
19	Active	38	48	52	1	0	0	2	1	2	10	0	0	−76	−38	−66
20	Active	45	44	39	0	0	0	0	0	0	0	0	0	6	16	21
21	Sham	44	37	37	1	1	2	1	2	3	0	0	0	−9	−14	−19
22	Sham	35	30	34	1	1	1	1	1	0	0	0	0	2	10	13
23	Sham	50	49	55	1	1	2	1	0	2	0	0	0	−20	1	−35
24	Sham	59	55	52	3	1	3	3	3	3	0	0	0	−70	−76	−79
25	Sham	40	30	32	0	0	0	0	0	0	0	0	10	7	−36	−68
26	Sham	53	49	47	0	0	0	0	0	0	0	0	0	0	11	11
27	Sham	49	47	36	0	0	0	0	1	2	0	0	0	11	0	0
28	Sham	39	35	30	1	1	1	0	1	1	0	0	0	10	4	10
29	Sham	39	41	38	1	0	0	0	0	0	0	0	0	12	10	22
30	Sham	26	21	33	0	0	0	0	0	0	0	0	0	34	31	20
31	Sham	42	44	40	1	1	1	1	1	0	0	0	0	−2	−12	0
32	Sham	58	51	53	1	0	1	2	1	1	10	0	0	−146	−30	−18
33	Sham	33	36	37	0	0	0	0	0	0	0	0	0	14	19	20
34	Sham	46	36	28	0	0	0	0	0	0	0	10	20	0	−69	−173
35	Sham	35	32	30	0	0	0	1	1	0	0	0	10	9	−59	−78
36	Sham	48	48	43	1	1	2	4	0	0	0	0	0	−40	−1	−20
37	Sham	46	43	35	1	0	3	1	0	0	0	0	0	−20	7	−12
38	Sham	60	60	54	2	1	1	2	1	1	0	10	0	−40	−89	−49
39	Sham	51	41	35	2	1	2	2	2	1	0	10	10	−47	−91	−70
40	Sham	60	50	46	2	2	2	1	0	1	0	0	0	−20	−17	−43

**Table 2:** SURG-TLX scores.

	Mental Demand			Physical Demand			Temporal Demand			Task Complexity			Situational Stress			Distractions		
Subject	Pre	Online	Post	Pre	Online	Post	Pre	Online	Post	Pre	Online	Post	Pre	Online	Post	Pre	Online	Post
1	15	4	0	15	20	0	55	55	0	14	21	0	0	0	0	4	2	35
2	18	60	52	40	44	65	48	24	30	20	16	9	8	0	6	2	9	4
3	36	44	80	12	36	20	80	39	27	26	10	18	64	48	30	0	0	0
4	15	16	3	22	8	4	7	1	1	20	35	25	40	9	4	0	0	0
5	68	28	48	32	51	26	95	7	45	12	60	18	39	0	0	0	80	4
6	56	33	48	85	44	24	22	0	8	11	22	60	42	8	33	0	70	0
7	10	20	36	11	40	65	28	7	0	20	45	10	20	30	20	6	0	52
8	18	12	15	16	6	8	8	3	4	70	40	45	16	18	24	0	0	0
9	12	12	21	8	0	39	64	20	39	0	16	0	70	55	45	6	3	7
10	45	51	10	24	6	8	40	24	6	51	0	4	11	34	2	0	80	0
11	48	16	15	12	21	9	39	50	24	7	8	5	70	4	55	0	36	0
12	14	2	5	30	18	9	13	12	30	80	12	12	44	30	28	0	0	0
13	3	2	2	12	8	40	68	48	56	36	24	10	39	24	42	0	0	0
14	18	6	2	7	8	4	40	6	8	20	12	15	60	20	6	0	0	0
15	30	24	52	20	24	21	2	3	2	60	32	15	48	32	12	0	0	0
16	24	52	8	50	32	45	3	0	2	28	14	6	56	10	6	0	12	0
17	28	40	30	14	40	50	80	10	4	64	20	30	36	4	3	0	0	16
18	56	33	10	18	6	36	12	0	8	36	48	50	64	28	10	0	36	0
19	36	40	48	4	3	3	6	18	6	39	52	65	75	20	9	0	0	0
20	80	48	40	0	0	10	36	36	15	12	10	5	60	44	40	6	6	0
21	12	36	55	27	24	36	24	22	20	35	21	36	52	0	7	0	36	0
22	33	36	36	5	3	10	60	32	48	22	20	33	55	45	44	0	0	0
23	22	16	30	52	55	55	36	30	36	0	6	2	75	56	20	2	0	0
24	21	30	28	32	44	39	24	28	24	80	55	55	6	5	6	0	0	0
25	65	75	56	24	11	22	0	0	12	42	24	52	52	56	44	0	0	0
26	65	14	0	2	2	4	16	2	4	52	32	15	39	6	10	0	5	4
27	33	8	9	0	1	6	12	20	12	50	24	16	10	0	0	3	4	2
28	3	20	26	0	0	0	3	21	24	28	24	16	60	60	45	24	24	36
29	12	15	2	8	4	6	50	0	3	30	4	0	44	4	2	0	4	5
30	20	10	10	9	0	9	48	56	56	30	27	8	70	39	42	0	40	60
31	36	40	12	4	20	7	24	21	21	22	18	28	60	44	35	0	0	0
32	48	12	36	10	16	21	75	14	14	48	60	65	16	6	3	0	0	0
33	2	5	30	36	25	40	28	27	3	15	24	21	21	12	15	0	0	0
34	45	75	68	68	30	48	16	12	15	8	36	21	60	48	2	0	0	2
35	56	48	56	8	7	9	2	0	4	90	60	75	48	16	33	0	21	0
36	0	0	0	55	36	35	6	5	3	40	21	10	24	8	12	30	45	20
37	30	30	55	12	21	32	2	7	2	20	3	10	14	10	9	0	33	0
38	14	8	12	4	18	7	60	56	56	42	36	33	85	85	80	0	0	0
39	6	0	0	0	0	0	30	10	15	12	6	6	24	12	8	0	0	0
40	24	26	32	3	3	3	54	68	64	90	72	80	56	48	39	0	0	0

**Table 3:** GLMM outputs.

lme4	Estimate	Std. Error	t value	p-value
**Performance Score**				
Intercept	4.88768	0.29082	16.807	< 0.001
Group	0.29698	0.18388	−1.615	0.106
Block	0.50025	−0.08437	−5.929	< 0.001
Group:Block	0.26581	0.05330	4.987	< 0.001
**Time**				
Intercept	2.94008	0.17666	16.643	< 0.001
Group	0.06140	0.11177	0.549	0.583
Block	0.16068	0.04052	3.965	< 0.001
Group:Block	−0.03349	0.02562	−1.307	0.191
**Error**				
Intercept	2.26817	0.35441	6.400	< 0.001
Group	−0.29501	0.22370	−1.319	0.187
Block	−0.57666	0.10759	−5.360	< 0.001
Group:Block	0.31449	0.06768	4.647	< 0.001

**Post-hoc comparison p-values across blocks:**				
	PS	Time	Error	
**Active tDCS**				
Pre vs. Online	< 0.001	< 0.001	< 0.001	
Pre vs. Post	< 0.001	< 0.001	< 0.001	
Online vs. Post	0.089	0.042	0.174	
**Sham tDCS**				
Pre vs. Online	0.750	0.002	0.887	
Pre vs. Post	0.688	< 0.001	0.525	
Online vs. Post	0.265	0.164	0.259	

**Post-hoc comparison p-values between interventions:**				
Active vs Sham	PS	Time	Error	
Pre	0.868	0.756	0.904	
Online	0.163	0.895	0.092	
Post	0.002	0.735	< 0.001

**Table 4:** Supporting Analyses with Friedman Test for differences across blocks (pre vs. online vs. post) and Mann Whitney U Test for differences between groups (active vs. sham).

	Time		Error		Performance score	
	Active	Sham	Active	Sham	Active	Sham
Friedman Test						
Degrees of freedom	2	2	2	2	2	2
test statistic	17.636	14.831	4.578	0.019	13.636	1.013
p-value	< 0.001	0.001	0.10	0.99	0.001	0.603
Post-hoc comparison p-value						
Pre vs. Online	0.066	0.043	–	–	0.053	–
Pre vs. Post	< 0.001	0.001	–	–	0.001	–
Online vs. Post	0.207	0.618	–	–	0.707	–

	Time		Error		Performance Score	
Mann Whitney U Test - Active vs. Sham (p-value)						
Pre	0.86		0.68		0.76	
Online	0.97		0.96		0.09	
Post	0.80		0.007		0.005

**Table 5:** Further Sub-Analyses of skill levels and initial vs. later knots.

a) Low and High Skill performance scores. Values are medians.						
	Low Skill			High Skill		
	Active	Sham	p-value	Active	Sham	p-value
Pre	−13.0	−30.0	0.425	8.5	9.5	0.940
Online	−5.5	−15.5	0.345	15.5	7	0.150
Post	4.5**	−27.5	0.023	20.5	10.5	0.028


b) Initial knots vs Last knots performance scores. Values are medians						
	Active			Sham		
	Initial Knots	Last Knots	p-value	Initial Knots	Last Knots	p-value
Pre	−12.3	0.8	0.191	−54.8	−1.0	0.001
Online	−2.0	3.3	0.247	−6.8	−18.3	0.040
Post	5.0	16.8	0.142	−24.0	−19.5	0.446

Asterix indicates significant difference from pre-. ** = *p* < 0.01.

**Table 6:** Sensations reporting.

	Proportion of participants			Sensation severity ranking		
	Active (*n* = 20)	Sham (*n* = 20)	p-value^a^	Active	Sham	p-value^b^
Itching	12	10	0.75	1.25 (1.21)	0.85 (1.14)	0.29
Pain	7	7	> 0.99	0.55 (0.89)	0.60 (0.99)	0.87
Burning	14	12	0.74	1.05 (1.05)	1.25 (1.41)	0.61
Warmth	13	9	0.34	1.00 (1.70)	0.80 (1.15)	0.59
Pinching	11	7	0.34	0.70 (0.80)	0.40 (0.60)	0.19
Metallic taste	0	0	> 0.99	0.00 (0.00)	0.00 (0.00)	> 0.99
Fatigue	4	3	> 0.99	0.30 (0.66)	0.25 (0.72)	0.82

Participant reported sensation proportions and mean severity ranking (SD).

tDCS = transcranial direct‐current stimulation.

a Fisher's exact test.

b Independent sample t-test.

## 2 Experimental Design, Materials and Methods

### 2.1 Participants

**Fig. 1:**
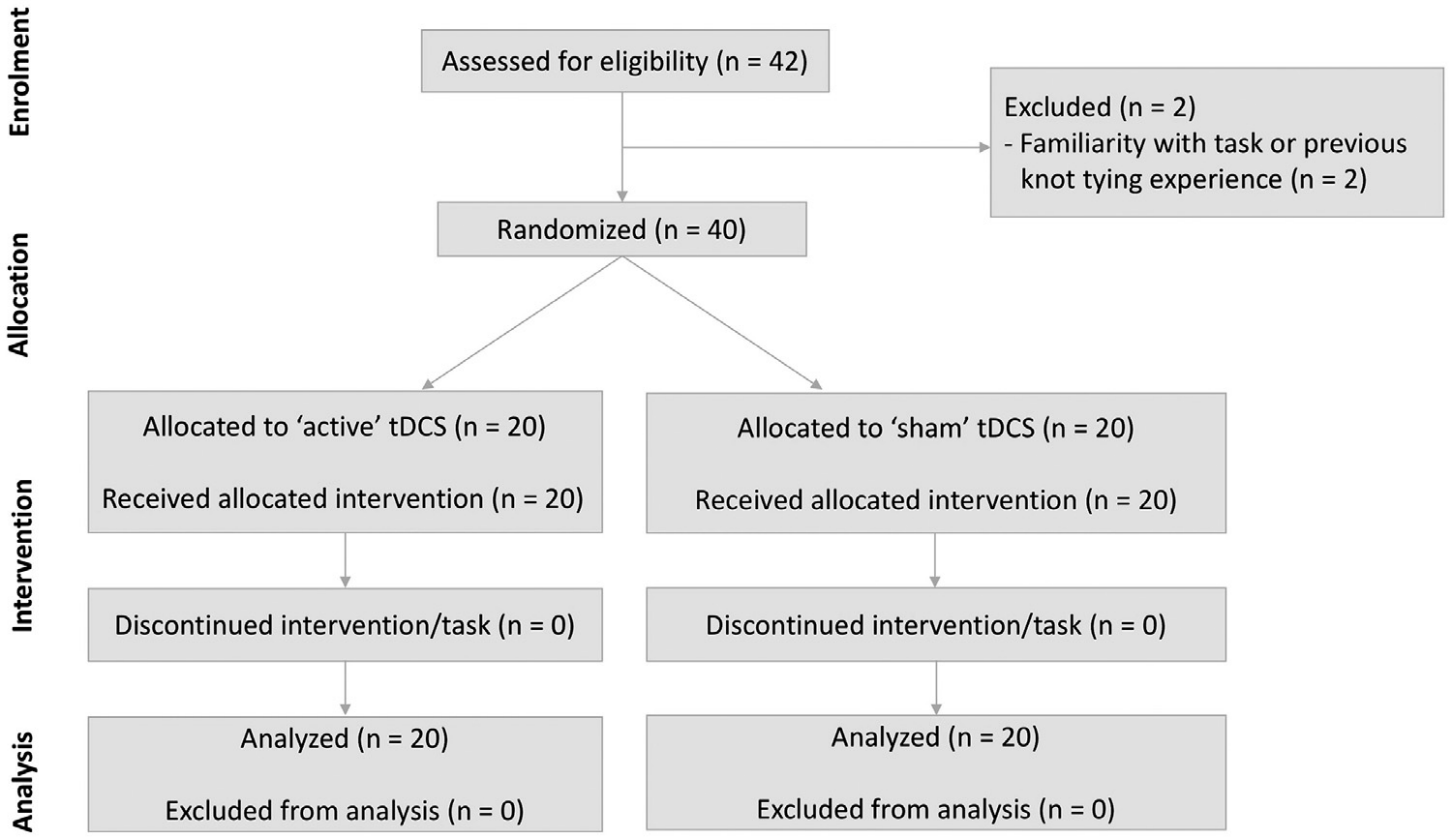
Enrolment, randomization, and analysis of study participants.

42 healthy medical students were recruited and information on demographics, handedness and open knot-tying experience was obtained. Participants reporting prior knot-tying ability were screened through a demonstration of their knot-tying ability. Agreement was required between two assessors (RP and JA) that no advantageous knot-tying familiarity or else participants were excluded (*n* = 2) (Fig. 1). Participants were additionally screened and excluded if they reported a history of traumatic head injury, neuropsychological condition, metallic implants, or adverse events to neurostimulation (*n* = 0).

### 2.2 Study design

Participants were randomly allocated using an online random number generator to receive a single 15 min session of either active (*n* = 20, 9 males, 11 females, mean (SD) age 21.3 (2.5) years) or sham (*n* = 20, 9 males, 11 females, mean (SD) age 21.9 (2.2) years) tDCS in a 1:1 ratio. In an initial one-hour training session, each participant was trained to perform open one-handed reef knots on a commercial bench-based knot tying trainer (Limbs & Things Ltd, Bristol, UK). Training was uniformly delivered firstly with 50 min of structured guidance using a dual-colored cord and a stepwise approach of observation only, observation with direction, performance under direction, and stand-alone performance. Participants were then allowed 10 min to familiarize themselves with the task using a 2–0 Polysorb Vicryl suture (Medtronic Ltd, Watford, UK). Following training, participants were screened for sufficient competency and instructed to tie three-throws of a surgical reef knot to determine development of to proceed to task assessment. Each participant then completed three separate blocks of a knot-tying task pre-tDCS, online-tDCS and post-tDCS, with each block separated by a 10-minute rest. All participants and the investigator administering participant training and measuring outcomes (JA) were blinded to the mode of stimulation. Knowledge of randomization order and group allocation was limited to one investigator (RP) who was restricted from data access or data-analysis.

### 2.3 Transcranial direct-current stimulation

During active tDCS, a 15-minute session of 2.0 mA stimulation was delivered using a Soterix Platform 1 × 1 Low Intensity Smart Scan™ tDCS device (SOTERIX MEDICAL INC, New York, NY). A pair of 0.9% saline-soaked 35cm^2^ electrodes were placed over the bifrontal prefrontal cortex (PFC). The anode and cathode were placed over the left and right PFC (F3 and F4 in the 10/20 electrode system) respectively [8] and affixed by a single circumferential strap. During sham tDCS, stimulation involved only a 30-second ramp up to 2.0 mA followed by an immediate ramp down, which is a method of effective blinding in tDCS studies [9].

### 2.4 Surgical task

Within each block, a 15-minute surgical task was performed which required nine repetitions of an open reef knot (3 throws per knot). Participants placed each knot on a commercial knot-tying bench rig over 5 mm pre-marked areas (Figure 2a), as described in a validated proficiency-based knot-tying curriculum [10,11]. A maximum cutoff time of 60-seconds (s) was allowed for each knot, with 30 s inter-knot rest periods. No feedback or reinforcement was given after the initial training period or during the task sessions.

### 2.5 Technical skill assessment

To assess knot-tying performance, an adapted performance score (PS) was determined based on a prior validated calculation [10,11]. This was based on time and error subcomponents to ensure that both speed and accuracy were accounted for:

PS (arbitrary units, au) = maximum cutoff time (60 s) - [completion time - (10 x error total)]

Error total was made up of the following subcomponents:

• Accuracy: distance (mm) between the suture and the colored target segments
• Gap: distance (mm) between the final knot and rubber tubes.
• Slippage (au): 10 error points were applied if the knot slipped by more than 3 mm. 20 error points were applied if the knot unraveled
• Breakage (au): 20 error points were applied if the suture thread broke on assessment

Although Scott and colleagues [10,11] allocated a score of zero to any negative values obtained by their participants, we report the actual scores including any negative values scored by our participants. This adaptation was considered necessary as our students were inexperienced with the task and thus likely to obtain negative scores.

### 2.6 Subjective workload and sensations

After each block, participants completed a SURG-TLX (Surgery Task Load Index) questionnaire (Supplementary material: DOI: 10.14469/hpc/7890) [12]. This comprises six subscales: mental demand, physical demand, temporal demand, task complexity, situational stress and distractions. The participant scores each domain on a weighting scale (scoring range: 0–5) and a visual analogue scale (scoring range: 0–20), which are multiplied to obtain final scores (maximum=100, minimum=0). A high score indicates that the participant felt this domain notably impacted their perceived workload, whilst a low domain score reflects the opposite. Following the active and sham online-tDCS blocks, all participants completed a four-point scale questionnaire (Supplementary material: DOI: 10.14469/hpc/7890) [13] on side-effects requiring severity to be ranked from 0 (none) to 4 (strong).

### 2.7 Data-analysis

The chi-squared test was used to analyze baseline demographics and estimation of intervention. The Shapiro-Wilk test determined that all outcomes measures were non-parametric. For behavioral performance assessment, PS and its subcomponents (time and error) were analyzed using a generalized linear mixed model (GLMM) for interaction and main effects of group and block, with participant as a random effect. To meet the requirements of a Gamma distribution, data transformations were first required. To facilitate this, an individual's PS was subtracted from the highest PS value in the dataset. Models were compared using the Akaike information criterion (AIC) with the smallest AIC retained. Multiple pairwise comparisons were corrected using Tukey's post-hoc test. A further supporting analysis was conducted to corroborate the GLMM findings. Firstly, a Friedman's test was used to analyze differences across the blocks in each group (pre vs. online vs. post). Secondly, the Mann-Whitney U test was used to identify differences between the intervention groups (active vs. sham) at each time point.

Further analyses were performed, firstly to stratify participants into ‘low-skill’ and ‘high-skill’ subgroups which was based on their individual baseline (pre-tDCS) performance score (‘low-skill’ = bottom 50% of dataset; ‘high-skill’ = top 50% of dataset). The Friedman test was again used to analyze differences in subgroup PS and overall SURG-TLX scores over the three blocks. The Wilcoxon signed-rank test with Bonferroni correction was used for post-hoc comparisons. Similarly, the Mann-Whitney U test was again used for analysis of subgroup PS and SURG-TLX scores between the active and sham groups during each block. Furthermore, to reveal if there was any variation in performance within the task blocks themselves, a comparison of the initial four and last four knots in each block was performed using the Wilcoxon signed-rank test. Proportions and severity rankings of sensations between the active and sham groups were compared using the Fisher's exact test and independent *t*-test. For all tests including after correcting for post-hoc comparisons, a p-value of <0.05 was considered statistically significant. Analysis was performed using the lme4 package in R v.3.6.3 (The R Foundation for Statistical Computing, Vienna) and SPSS v.25.0 (IBM Corp, Armonk, NY).

## Ethics Statement

Participants were recruited following Imperial College Research Ethics Committee approval (18IC4706) and written informed consent was obtained from all subjects.

## CRediT Author Statement

**Ronak Patel:** Conceptualization, Methodology, Resources, Formal analysis, Writing - review & editing; **Harsimrat Singh:** Conceptualization, Methodology, Funding acquisition, Writing - review & editing, Supervision; **James Ashcroft:** Conceptualization, Methodology, Resources, Investigation, Funding acquisition; **Adam J Woods:** Conceptualization, Methodology, Writing - review & editing; **Ara Darzi:** Funding acquisition, Supervision; **Daniel R Leff:** Conceptualization, Methodology, Funding acquisition, Writing - review & editing, Supervision.

## Declaration of Competing Interest

The authors declare that they have no known competing financial interests or personal relationships which have or could be perceived to have influenced the work reported in this article.
